# ﻿Phlebotomine sand flies (Diptera, Psychodidae) from Spain: an updated checklist and extended distributions

**DOI:** 10.3897/zookeys.1106.81432

**Published:** 2022-06-17

**Authors:** Daniel Bravo-Barriga, Ignacio Ruiz-Arrondo, Rosa Estrada Peña, Javier Lucientes, Sarah Delacour-Estrella

**Affiliations:** 1 Universidad de Extremadura, Facultad de Veterinaria, Departamento de Sanidad Animal, Parasitología, Avda. Universidad s/n, 10003 Cáceres, Spain Universidad de Extremadura Cáceres Spain; 2 Center for Rickettsiosis and Arthropod-Borne Diseases, Hospital Universitario San Pedro-CIBIR, Logroño, Spain Hospital Universitario San Pedro-CIBIR Logroño Spain; 3 Animal Health Department, The AgriFood Institute of Aragon (IA2), School of Veterinary Medicine, University of Zaragoza, 50013 Zaragoza, Spain University of Zaragoza Zaragoza Spain; 4 Departamento de Investigación y Desarrollo (I+D). Quimera. B.S. Calle Olivo, 14, 50016 La Puebla de Alfindén, Spain Departamento de Investigación y Desarrollo La Puebla de Alfindén Spain

**Keywords:** Catalogue, *Leishmania*, phlebovirus, *Phlebotomus*, sand fly–borne viruses, *Sergentomyia*, spatial distribution, taxonomy

## Abstract

Phlebotomine sand flies (Diptera: Psychodidae) are the natural vectors of *Leishmania* spp. (Kinetoplastida: Trypanosomatidae) and phleboviruses (Bunyavirales: Phenuiviridae). In Spain, these vectors appear to be increasing their geographical distribution and have serious repercussions on public and veterinary health, encouraging studies of sand flies and their associated pathogens. An up-to-date and easily accessible compendium of current and historical data on their presence and detailed distribution is a crucial step towards the development and implementation of appropriate preventive strategies. A checklist on the presence and distribution of sand flies in Spain is compiled from data extracted from a comprehensive review of scientific literature published between 1909 and 2021 and our new records on the presence of sand flies specimens collected under the entomological surveillance of bluetongue vectors from the Spanish Ministry of Agriculture, Fishery and Food (MAPA) during the period 2004–2021. In total, 13 Spanish species of sand flies (two of them with controversial status) belonging to two genera and six subgenera are presented in this updated checklist, including new distribution data for seven species, among which several stand out as confirmed or suspected vectors of *Leishmaniainfantum*: *Phlebotomusariasi*, *Ph.langeroni*, *Ph.mascittii*, *and Ph.perniciosus*.

## ﻿Introduction

Phlebotomine sand flies are a major public and veterinary health concern due to their haematophagous habits that allow these insects to be natural vectors of *Leishmania* spp. (Kinetoplastida: Trypanosomatidae), arboviruses (phlebovirus, vesiculovirus, and orbivirus) (Akhoundi et al. 2016; Ayhan and Charrel 2017) and, in South America, also the bacterium *Bartonellabacilliformis* (Sánchez Clemente et al. 2012). In Europe in recent years, the density of sand flies has increased in endemic areas or has spread into new areas (Medlock et al. 2014), causing progressively more autochthonous outbreaks of phlebotomine-borne diseases (González et al. 2017; García San Miguel et al. 2021). More than 50 species of *Phlebotomus* Loew, 1845 have been described in Europe, North Africa, the Middle East, and the Caucasus, and eleven of them are implicated in the transmission of pathogens (Alten et al. 2016). Updated studies on taxonomic, spatiotemporal, and bio-ecological aspects, as well as the epidemiological status, of sand flies are crucial to develop effective entomological surveys and control plans. Furthermore, a global review of the information available can be useful in detecting regions lacking data. For easier access, all this information must be compiled, ordered, and updated, allowing effective management for students, professors, general researchers, medical and veterinary entomologists, animal and public health authorities, and public and private institutions involved in the study and control of sand flies and their related pathogens.

The first report of sand flies in Spain dates back to 1909 (Czerny and Strobl 1909) when a female of *Phlebotomusariasi* Tonnoir, 1921 was found in Madrid but mistakenly reported as *Phlebotomuspapatasi* (Scopoli, 1786). Other females from the same sample were not identified, but dry-preserved and assumed to be identical to the first, until León Sanz et al. (1998) analyzed them, adding more details to the document by Czerny and Strobl (1909), identifiying more females of *Ph.ariasi* and *Phlebotomusperniciosus* Newstead, 1911.

Most of the studies on the presence and phenology of sand flies in Spain are concentrated between the 1970s to 1990s, where authors such as Francisco Morillas-Márquez (Morillas-Márquez et al. 1982a, b, 1983a, 1991), Ezequiel Martínez-Ortega (Martínez-Ortega et al. 1982; Martínez-Ortega 1986; Martínez-Ortega and Conesa Gallego 1987a), Jean-Antoine Rioux (Rioux et al. 1974, 1975, 1984), Montserrat Gállego (Gállego et al. 1990) and Javier Lucientes (Lucientes-Curdi et al. 1991, 1995) improved the distributional knowledge, the biology, their epidemiological role, and also the description of two new species (Úbeda Ontiveros et al. 1982; Morillas-Márquez et al. 1983b).

Gil Collado et al. (1989) carried out a review of the distribution, morphology, and biology of sand flies in Spain and described eleven species. Later, Gállego Berenguer et al. (1992) updated these data on the distribution of sand flies in the northeast of the Iberian Peninsula and the Balearic Islands. Since then, one new species has been described (Depaquit et al. 1998), another was reported for the first time for Spain (Martínez-Ortega et al. 1996), and corrections were made in the identification of historical sand fly collections (León Sanz et al. 1999).

In recent years, further investigations have been initiated mainly focusing on the role of these insects as vectors of *Leishmania* spp. and phleboviruses (Sanbonmatsu-Gámez 2005; Barón et al. 2011; Alcover et al. 2014; Ballart et al. 2014; Bravo-Barriga et al. 2016; Remoli et al. 2016), especially as a result of the largest outbreak of human leishmaniosis in Madrid in 2009 (Jiménez et al. 2013; González et al. 2017, 2021). In light of this new situation, the geographical and epidemiological status of phlebotomine knowledge in our country has been substantially improved.

The aim of this study is to update the list of sand flies present in Spain by compiling the distribution records by provinces contained in the bibliography, and to increase the information by adding our own entomological results carried out between 2004 and 2021 in all Spanish regions based on collections from the MAPA. The updated data provided will be useful for the design of new research, surveillance, and vector control programmes as well as the assessment of the risk of pathogens transmission by sand flies in Spain.

## ﻿Materials and methods

### ﻿Data collection

Knowledge of the distribution of Spanish sand fly species has been synthesised from two sources:

1. A comprehensive review of 136 scientific articles and grey literature (such as government reports, conference proceedings, graduate dissertations, and relevant MSc theses) published between 1909 and 2021. For doctoral theses written by article compendium, only their publications were taken into account. These materials were sourced through PubMed, ResearchGate, Scopus, Web of Science, Google Scholar, and digital repositories (e.g., Digital.CISC, TESEO, Dehesa, and Dialnet) using the following keywords in English, French, and Spanish: Phlebotominae, Phlebotomine, *Phlebotomus*, sandfly, sand flies, Phlébotomes, flebotomos, Spain, Espagne, España, *Leishmania*, leishmaniosis, leishmaniasis, and phlebovirus.

Some earlier materials that were difficult to obtain were provided courtesy of an exchange between experts. The retrieved papers form the basis for the checklist which was used to confirm species records and are thus dependent on the quality of the identification made by the authors at the time of publication of the record. The bibliographic references associated with each species recorded for Spain are presented in Suppl. material 1.

1. Unpublished entomological data of sand flies collected in traps for the monitoring of *Culicoides* biting midges (Diptera: Ceratopogonidae), vectors of the bluetongue virus (BTV). Since 2000, a national surveillance, control, and eradication of BTV programme has been carried out in Spain supported by the MAPA and coordinated by the University of Zaragoza (Zaragoza, Spain).

The surveillance data presented here are based on the analysis of 1179 sample points belonging to 1040 municipalities between 2004 and 2021 from almost all Spanish provinces except the autonomous cities Ceuta and Melilla. All islands of the Balearic Archipelago (Mallorca, Menorca, Ibiza, and Formentera) are considered as a single province; however, we do indicate the sand fly species when they are recorded for the first time in a specific island.

Each collection site was georeferenced using a Garmin GPS 12 Global Position Device with geographical coordinate system (EPSG: 23030-ED50/UTM zone 30N). CDC-UV traps (Miniature Blacklight trap 1212, John W. Hock Company, Gainesville, FL, USA) were placed overnight once a week throughout the year long in a variable number of animal holdings composed mainly of sheep, goats, and cattle. These traps collect not only *Culicoides* but also many other insects that exhibit a positive phototropism such as sand flies and mosquitoes, among others.

Captured specimens were stored in 70% ethanol and morphological identification was carried out following the characters described by Martínez-Ortega and Conesa Gallego (1987b) and Gállego Berenguer et al. (1992). Female specimens were identified by microscopic observation of the spermatheca, after dissection and slide mounting of the last three abdominal segments with Hoyer’s solution. Males were identified by direct stereomicroscopic observation of the features of the external genitalia.

The species included in this list are ordered alphabetically by subfamily, genus, and subgenus. Species names include authorities and year (Table 1). Nomenclaturally, we have used the organisation and abbreviations proposed by Rispail and Léger (1998) and Marcondes (2007) for the genera and subgenera of Phlebotominae. The subgenus Abonnencius proposed by Morillas-Márquez and Guevara Pozo (1994) for *Phlebotomusfortunatarum*Úbeda Ontiveros et al. (1982), an endemic species of the Canary Islands (Spain), has also been included. The status of some species in Spain is also briefly discussed in the Notes section.

**Table 1.** Checklist of sand flies species recorded in Spain, classified by genus and subgenus.

Genus	Subgenus	Species	Author/Year
*Phlebotomus*	*Abonnencius*	fortunatarum*	Úbeda Ontiveros, Morillas-Márquez, Guevara Benítez, López Roman & Cutillas Barrios, 1982
	*Larroussius*	*ariasi*	Tonnoir, 1921
		*langeroni*	Nitzulescu, 1930
		longicuspis*	Nitzulescu, 1930
		*perniciosus*	Newstead, 1911
	*Paraphlebotomus*	*alexandri*	Sinton, 1928
		*chabaudi*	Croset, Abonnenc & Rioux, 1970
		*riouxi* *	Depaquit, Killick-Kendrick & Léger, 1998
		*sergenti*	Parrot, 1917
	*Phlebotomus*	*papatasi*	(Scopoli, 1786)
	*Transphlebotomus*	mascittii*	Grassi, 1908
*Sergentomyia*	*Sergentomyia*	*fallax*	(Parrot, 1921)
		*minuta*	(Rondani, 1843)

*Species with Notes. The list of references used to generate the distribution map for each species is provided in Suppl. material 1.

### ﻿Current distribution

The distribution maps of each species have been made at province level (NUTS3) using the software QGIS Geographic Information System, version 3.22.0 (2021). The reference coordinate system established in the work was EPSG:4258-ETRS89. QGIS Association, http://www.qgis.org. (Figs 1–13).

Each figure shows the origin of the knowledge of the distribution of each species:

1. From data obtained from the literature review: Grey provinces for presence and white for absence of the species.
2. From the positive sampling points (black) of our entomological surveillance: If any of these sampling points is the first report for that province, that province is highlighted in green.

## ﻿Results

From the comprehensive bibliography reviewed, a total of 13 sand fly species have been reported in Spain (Table 1), although two of them have a controversial status (*Ph.longicuspis* Nitzulescu, 1930 *and Ph.riouxi* Depaquit, Killick-Kendrick & Léger, 1998) and are discussed in the Notes section. According to the nomenclatural criteria used, these 13 species belong to two genera and six subgenera, as follows: Phlebotomus (Abonnencius) (one species), Ph. (Larroussius) (four species), Ph. (Paraphlebotomus) (four species), Ph. (Phlebotomus) (one species), Ph. (Transphlebotomus) (one species), Sergentomyia (Sergentomyia) (two species).

**Figure 1.**
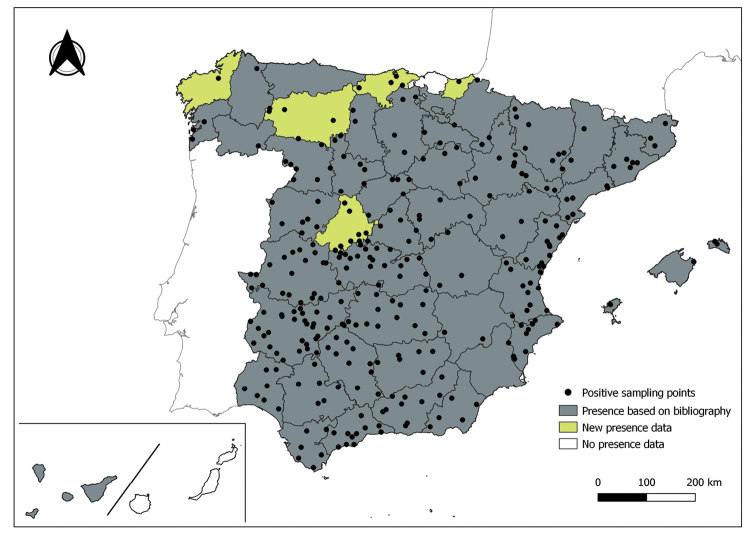
Distribution of *Phlebotomusperniciosus* in Spain.

**Figure 2.**
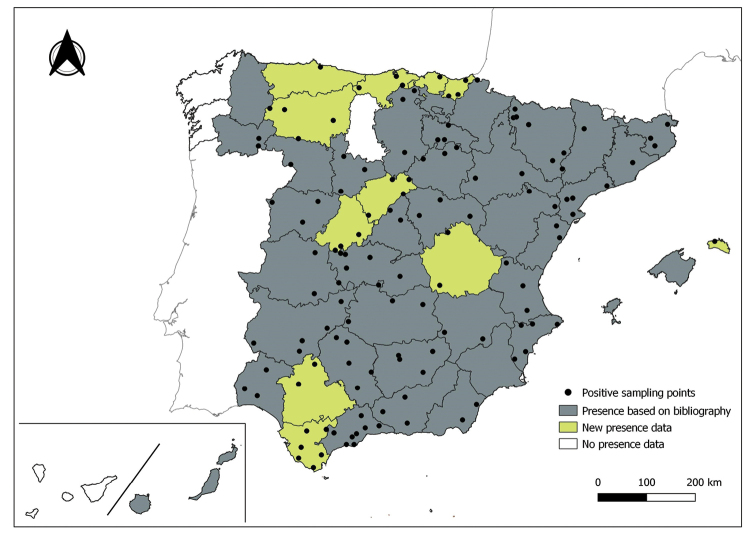
Distribution of *Phlebotomusariasi* in Spain.

**Figure 3.**
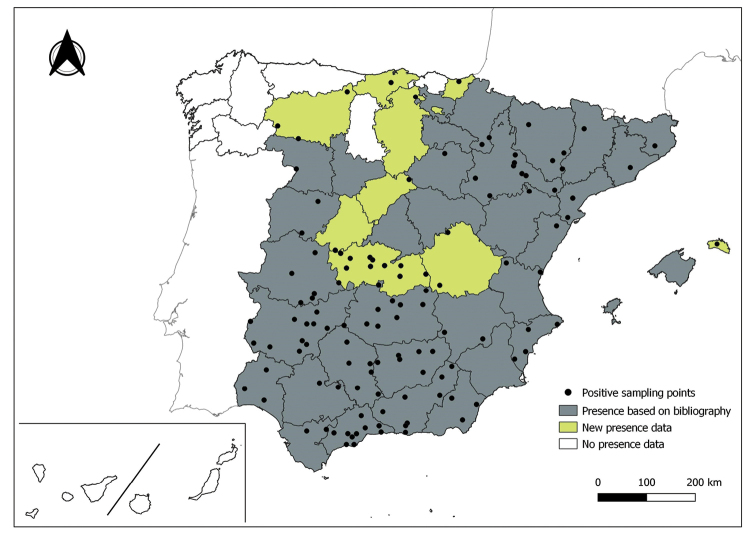
Distribution of *Phlebotomuspapatasi* in Spain.

**Figure 4.**
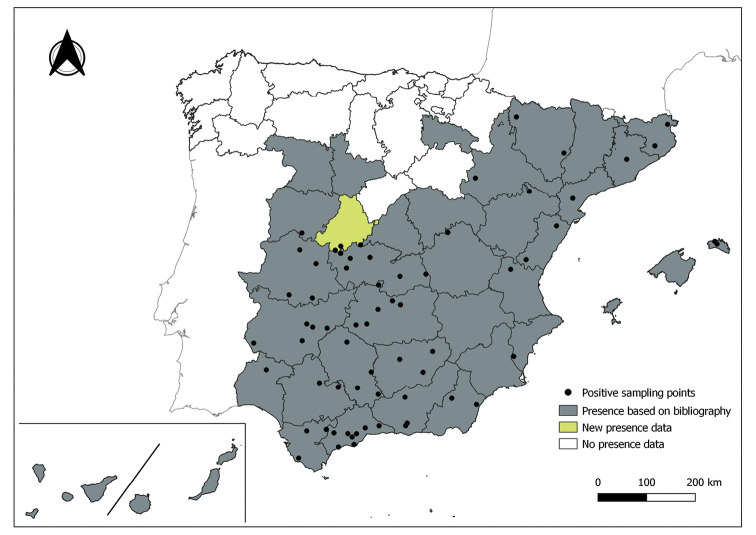
Distribution of *Phlebotomussergenti* in Spain.

**Figure 5.**
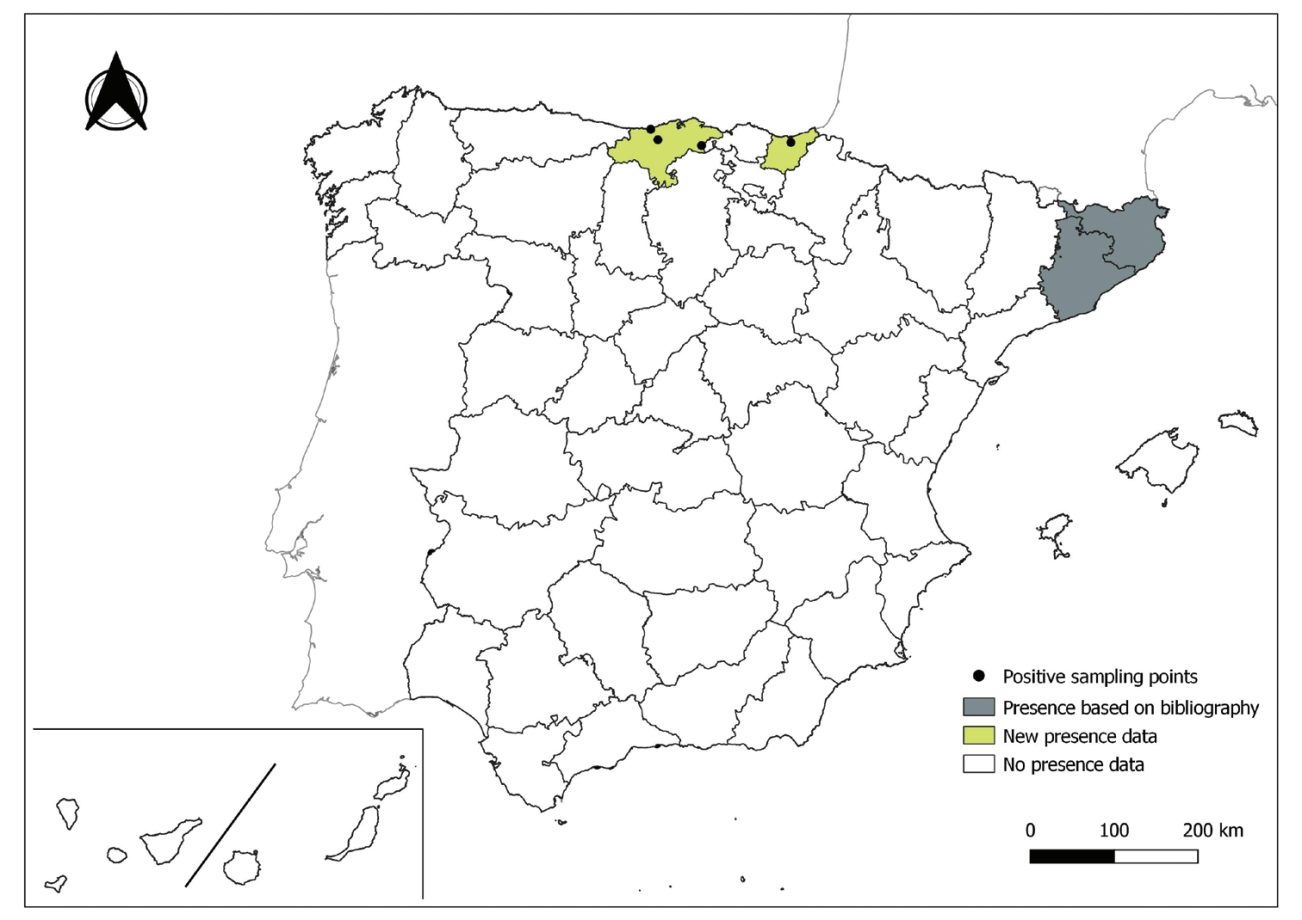
Distribution of *Phlebotomusmascittii* in Spain.

**Figure 6.**
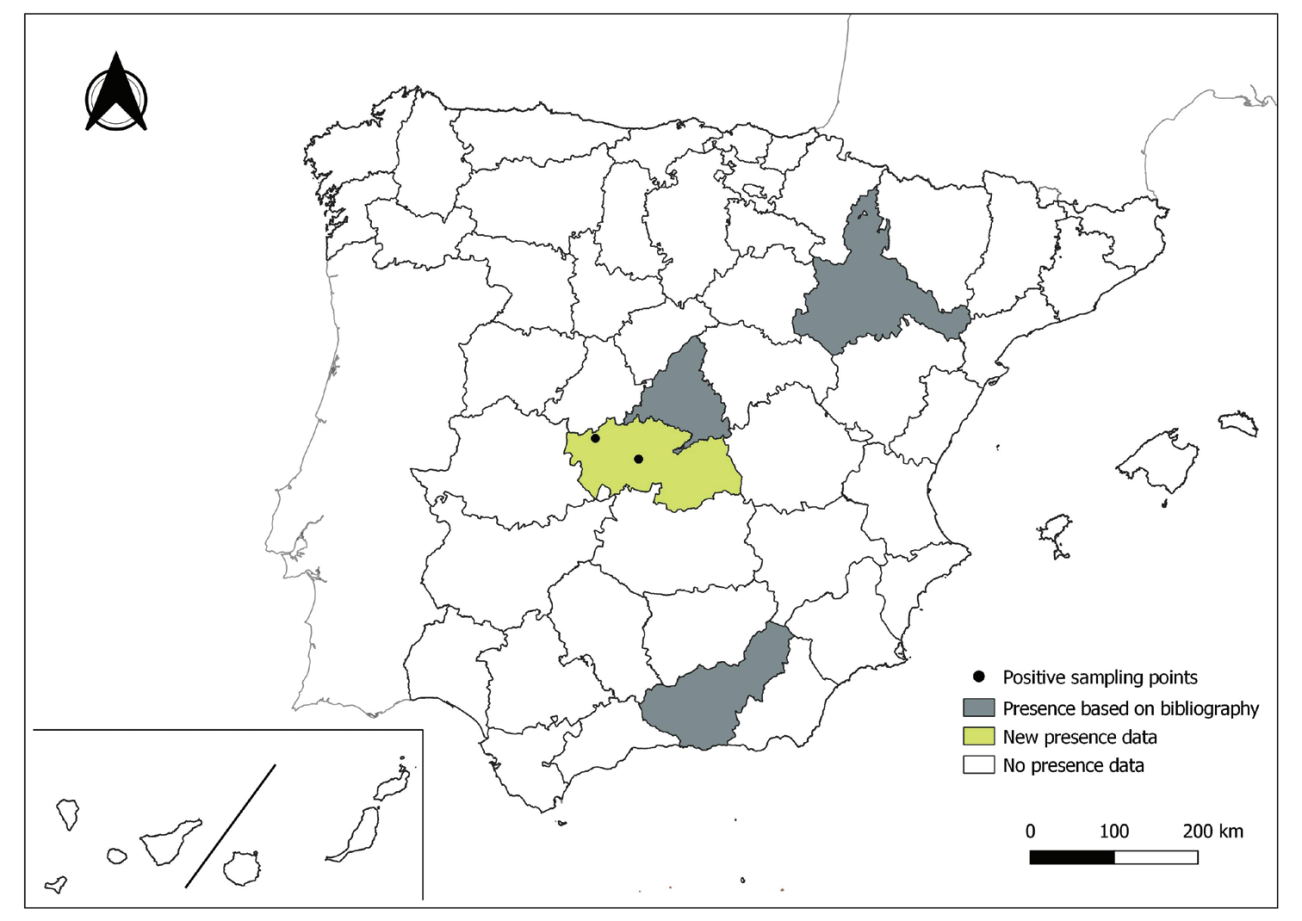
Distribution of *Phlebotomuslangeroni* in Spain.

**Figure 7.**
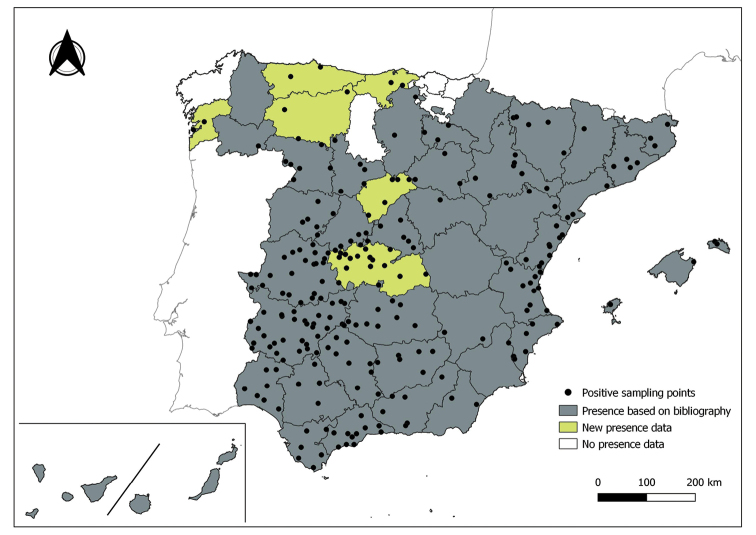
Distribution of *Sergentomyiaminuta* in Spain.

**Figure 8.**
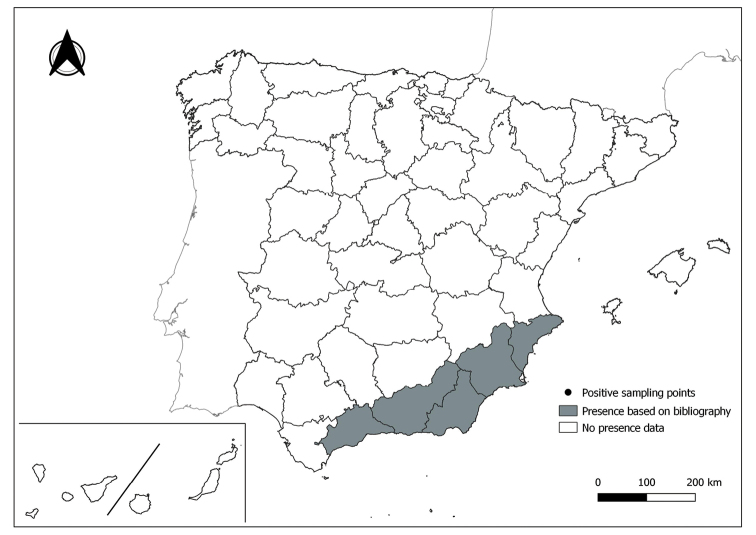
Distribution of *Phlebotomusalexandri* in Spain.

**Figure 9.**
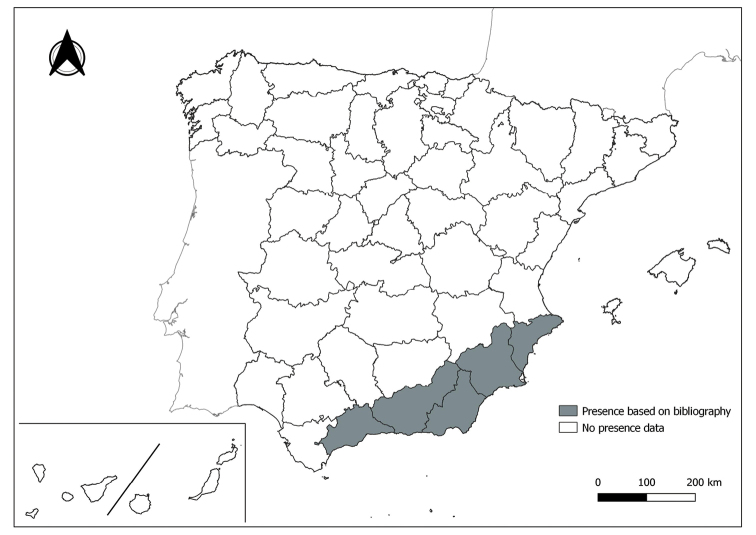
Distribution of *Phlebotomuschabaudi* in Spain.

**Figure 10.**
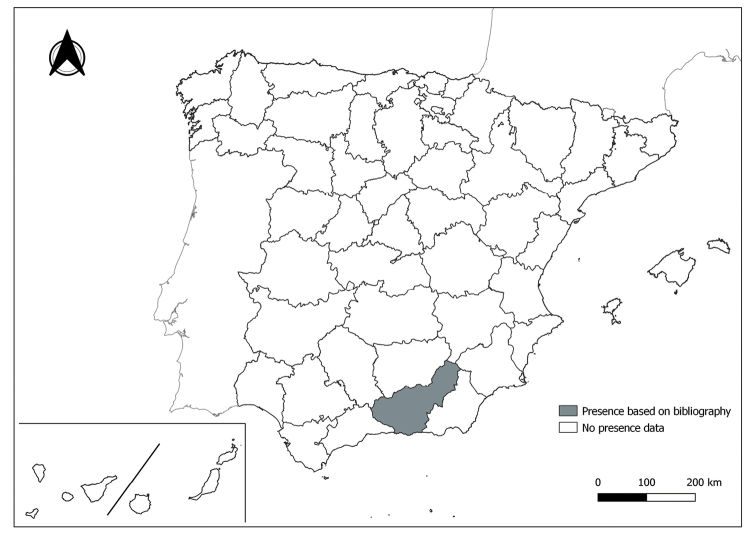
Distribution of *Phlebotomusriouxi* in Spain.

**Figure 11.**
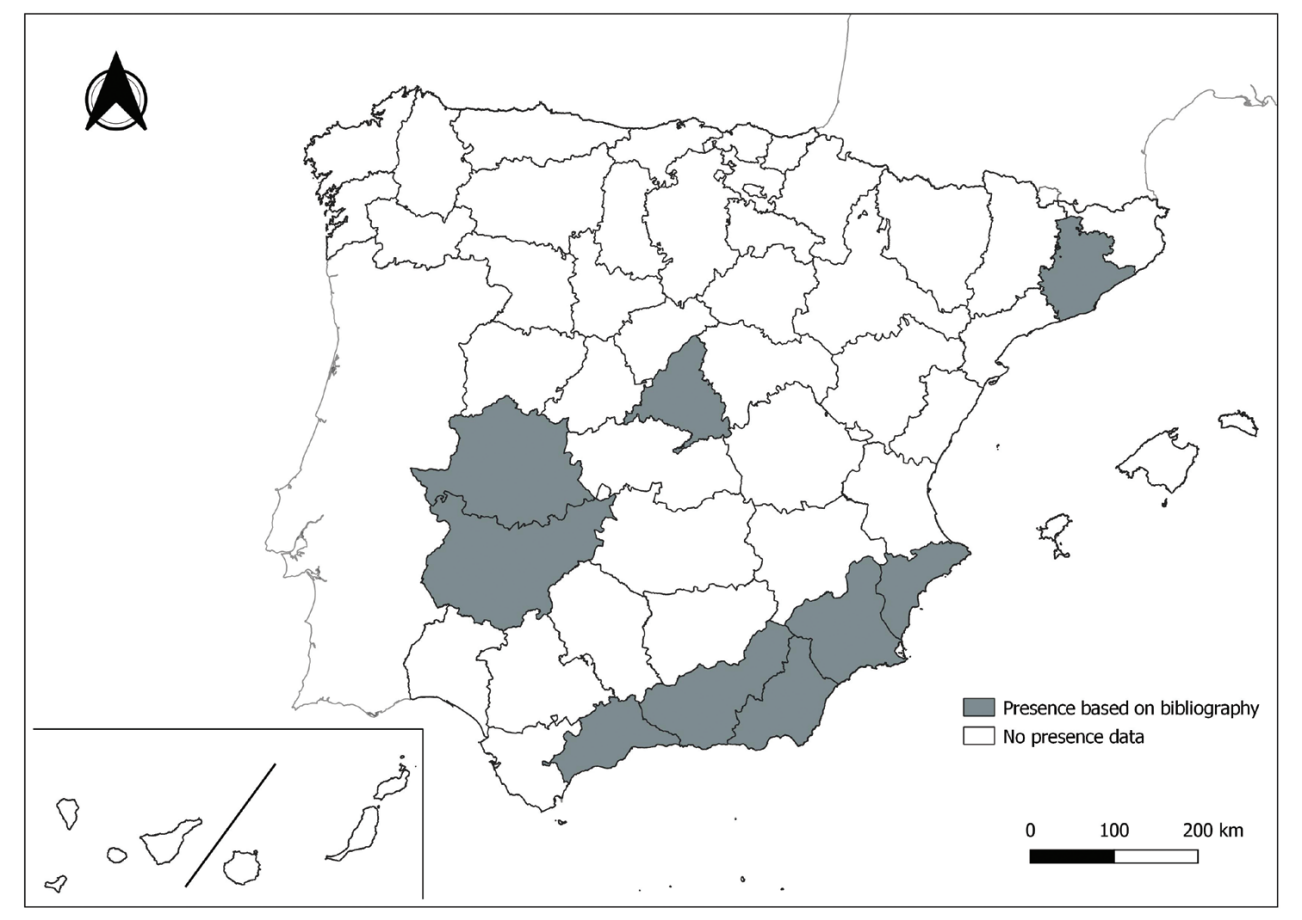
Distribution of *Phlebotomuslongicuspis* in Spain.

**Figure 12.**
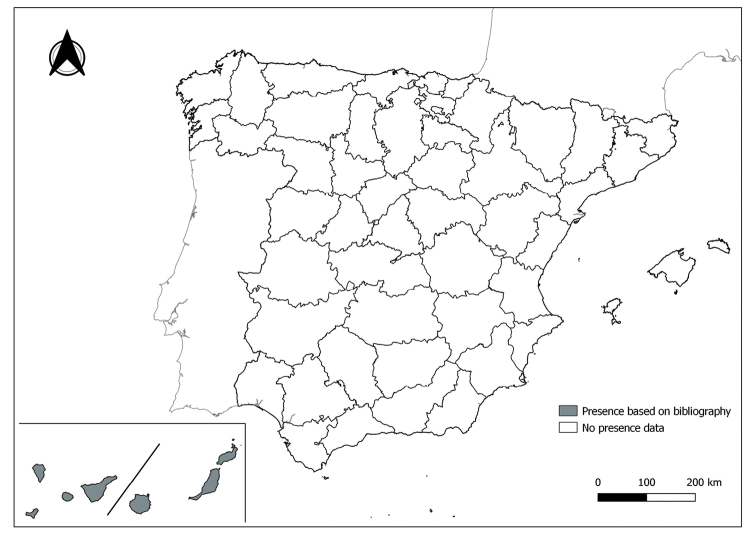
Distribution of *Sergentomyiafallax* in Spain. Note its absence from peninsula Spain.

**Figure 13.**
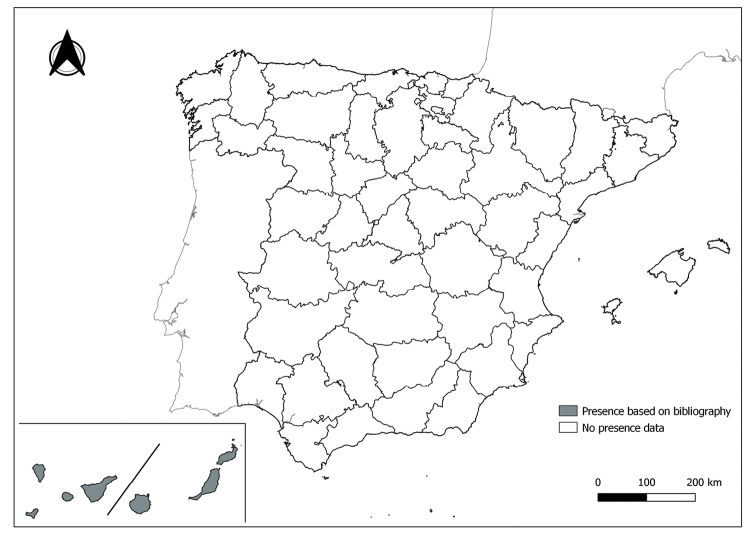
Distribution of *Phlebotomusfortunatarum* in Spain. Note its absence from peninsula Spain.

As a result of sand fly data collected between 2004 to 2021 as part of the entomological surveillance programme of *Culicoides* biting midges in Spain we record seven species of sand flies for the first time in some Spanish provinces, listed as follows. The distribution of the main *Leishmania* vectors is widened in five provinces for *Ph.perniciosus* (Fig. 1) and in ten provinces for *Ph.ariasi*, including the island of Menorca, for the first time (Fig. 2). *Phlebotomuspapatasi* is detected in eight new provinces in the centre and north of the country, as well as on the island of Menorca (Fig. 3). Regarding *Ph.sergenti* Parrot, 1917, a new province is cited but it is absent in the northwest of the Iberian Peninsula (Fig. 4). The distribution of *Phlebotomusmascittii* Grassi, 1908 is extended to two more provinces on the Cantabrian basin (Fig. 5). As for *Ph.langeroni* Nitzulescu, 1930, its presence is extended to one more province in the centre of the Iberian Peninsula (Fig. 6). Finally, the presence of the species *Se.minuta* (Rondani, 1843) is broadened to six more provinces (Fig. 7), being found in practically the whole country. It should be noted that, *Ph.alexandri* Sinton, 1928 (Fig. 8), *Ph.chabaudi* Croset, Abonnenc & Rioux, 1970 (Fig. 9), *Ph.riouxi* (Fig. 10), *Ph.longicuspis* (Fig. 11), *Se.fallax* (Parrot, 1921) (Fig. 12), as well as the endemic species of the Canary Islands, *Ph.fortunatarum* (Fig. 13), have not been detected in the course of the national entomological surveillance programme, maybe due to the use of biased sampling methods or specific trapping in ruminant farms.

### ﻿Notes

I. *Phlebotomusfortunatarum* is an endemic species from the Canary Islands (Spain), which was described for the first time in Gran Canaria island in 1982 (Úbeda Ontiveros et al. 1982), and later in other islands (Morillas-Márquez et al. 1984; Lane and Alexander 1988; Martínez-Ortega et al. 1988). Due to its morphological characteristics (Úbeda Ontíveros and Morillas-Márquez 1983), it could not be included in the subgenera already available, so the subgenus Abonnencius was proposed by Morillas-Márquez et al. (1984). However, Lane and Alexander (1988) rejected the subgenus Abonnencius and included *Ph.fortunatarum* in the subgenus Anaphlebotomus. Some years later, Morillas-Márquez and Guevara Pozo (1994) discussed and proved the validity of the subgenus Abonnencius, which according to the authors, should be retained until new complete classification proposed for the entire genus *Phlebotomus*.

II. *Phlebotomuslongicuspis* was first described in Tunisia as a variety of *Ph.langeroni* and was elevated to species status by Parrot (1936). This species is now considered common in North Africa (Pesson et al. 2004). Since its detection in Spain in 1982 by Morillas-Márquez et al. (1982b), numerous authors have cited the presence of this species based exclusively on male specimens in many regions, mainly in the south and east of the country (Martínez-Ortega et al. 1982; Rosado Maestre 1997; Blázquez Martín 1998; Martín-Sánchez et al. 2000). However, the morphological similarities of the copulatory structure with *Ph.perniciosus* have generated controversy about the validity of *Ph.longicuspis*. The difficulty of correctly determining the males of each species, together with numerous intermediate stages, have led authors such as Morillas-Márquez et al. (1991) and Collantes and Martínez-Ortega (1997) to conclude that this taxonomic criterion is not discriminatory. Nevertheless, Di Muccio et al. (2000) carried out a phylogenetic analysis on specimens of Phlebotomus species belonging to the subgenus Larroussius from Morocco using ITS2 rDNA sequences and suggested that they are a distinct species, despite slight morphological differences. In addition, isoenzyme studies and comparative DNA sequencing of a mitochondrial cytochrome b fragment (mtDNA) showed that some sympatric populations of *Ph.perniciosus* and *Ph.longicuspis* have the characteristics of a biological species (Pesson et al. 2004). Interspecific gene introgression and a new sibling species have been detected, making identification even more difficult. The proximity of Spain to North Africa increases the possibility of detecting specimens with intermediate characters (Collantes and Martínez-Ortega 1997). Old records based on morphology may not necessarily reflect the true geographical distribution or occurrence of *Ph.longicuspis* presented here for Spain. All records of *Ph.longicuspis* in Spain are from the 1980s and 1990s, when molecular tools were not used and the taxonomic identification of sand flies was based solely on morphological criteria. Currently, the Spanish sand fly specialist community assumes that records of *Ph.longicuspis* in Spain are all probably *Ph.perniciosus*. Therefore, we consider the presence of *Ph.longicuspis* in Spain uncertain, although we show its recorded distribution from the literature in Fig. 11. Actual genetic characterisation of more populations would be necessary to improve our knowledge and verify the current status of this species in Spain.

III. *Phlebotomusriouxi* was first described by Depaquit et al. (1998) based on specimens from Morocco, Tunisia, and Spain. *Phlebotomusriouxi* is a species closely related to *Ph.chabaudi*, with subtle morphological differences in some structures (Depaquit et al. 1998; Lehrter et al. 2017). Molecular studies on several populations from Algeria and Tunisia supported the validity of both *Ph.riouxi* and *Ph.chabaudi* as typological species (Bounamous et al. 2008; Boudabous et al. 2009; Lehrter et al. 2017). However, Tabbabi et al. (2014) proposed considering both species as synonyms after molecular analysis of specimens from a single locality in Tunisia. Thus, even if both species have been reported in Spain (Figs 9, 10), reservations remain because, despite regular works on sand flies in the province of Granada, *Ph.riouxi* has not been detected again. We consider the records of *Ph.riouxi* in Spain as *Ph.chabaudi* and therefore the presence of the former species is uncertain in Spain.

IV. *Phlebotomusmascittii* was first detected in the early 1980s in Barcelona and Girona (north-eastern Spain) (Rioux et al. 1984). However, since then it has not been found until our detection through the entomological surveillance of bluetongue vectors (Fig. 5). Furthermore, during a two-year (2019–2020) local research project aimed at revealing the diversity of bloodsucking dipteran pests in urban and rural areas of the Basque Country (northern Spain), specimens were detected in an urban cemetery. All of these new reports along the Cantabrian cornice (northern Spain) motivated the realisation of a study recently focused on this species, which delved into the distribution of its different haplotypes (Alarcón-Elbal et al. 2021).

## Supplementary materials

Supplementary material 1 List of bibliographic references associated with the distribution of sand flies by province in Spain Data type excel file. Explanation note The supplementary file includes the 136 references of scientific papers and grey literature listed in the checklist (ordered by ID, year and author). We also include the references of each species of sand fly by Spanish province. https://binary.pensoft.net/file/702898This dataset is made available under the Open Database License (http://opendatacommons.org/licenses/odbl/1.0/). The Open Database License (ODbL) is a license agreement intended to allow users to freely share, modify, and use this Dataset while maintaining this same freedom for others, provided that the original source and author(s) are credited.Daniel Bravo-Barriga, Ignacio Ruiz-Arrondo, Rosa Estrada Peña, Javier Lucientes, Sarah Delacour-Estrella

## References

[B1] Akhoundi M Kuhls K Cannet A Votýpka J Marty P Delaunay P Sereno D (2016) A historical overview of the classification, evolution, and dispersion of *Leishmania* parasites and sandflies. PLoS Neglected Tropical Diseases 10(3): e0004349. 10.1371/journal.pntd.0004349 PMC4777430 26937644

[B2] Alarcón-Elbal PM González MA Delacour-Estrella S Bravo-Barriga D Estrada Peña R Goiri F García-Pérez AL Lucientes J (2021) First findings and molecular data of *Phlebotomusmascittii* (Diptera: Psychodidae) in the Cantabrian Cornice (Northern Spain). Journal of Medical Entomology 58(6): 2499–2503. 10.1093/jme/tjab091 34021579

[B3] Alcover MM Ballart C Martín-Sánchez J Serra T Castillejo S Portús M Gállego M (2014) Factors influencing the presence of sand flies in Majorca (Balearic Islands, Spain) with special reference to *Phlebotomuspernicious*, vector of *Leishmaniainfantum*. Parasites & Vectors 7(1): 1–12. 10.1186/1756-3305-7-421 25192589 PMC4261246

[B4] Alten B Maia C Afonso MO Campino L Jiménez M González E Molina R Bañuls AL Prudhomme J Vergnes B Toty C Cassan C Rahola N Thierry M Sereno D Bongiorno G Bianchi R Khoury C Tsirigotakis N Dokianakis E Antoniou M Christodoulou V Mazeris A Karakus M Ozbel Y Arserim SK Erisoz Kasap O Gunay F Oguz G Kaynas S Tsertsvadze N Tskhvaradze L Giorgobiani E Gramiccia M Volf P Gradoni L (2016) Seasonal dynamics of phlebotomine sand fly species proven vectors of Mediterranean leishmaniasis caused by *Leishmaniainfantum*. PLoS Neglected Tropical Diseases 10(2): e0004458. 10.1371/journal.pntd.0004458 PMC4762948 26900688

[B5] Ayhan N Charrel RN (2017) Of phlebotomines (sand flies) and viruses: A comprehensive perspective on a complex situation. Current Opinion in Insect Science 22: 117–124. 10.1016/j.cois.2017.05.019 28805633

[B6] Ballart C Guerrero I Castells X Barón S Castillejo S Alcover MM Portús M Gállego M (2014) Importance of individual analysis of environmental and climatic factors affecting the density of *Leishmania* vectors living in the same geographical area: The example of *Phlebotomusariasi* and *P.perniciosus* in northeast Spain. Geospatial Health 8(2): 389–403. 10.4081/gh.2014.28 24893016

[B7] Barón SD Morillas-Márquez F Morales-Yuste M Díaz-Sáez V Irigaray C Martín-Sánchez J (2011) Risk maps for the presence and absence of *Phlebotomusperniciosus* in an endemic area of leishmaniasis in southern Spain: Implications for the control of the disease. Parasitology 138(10): 1234–1244. 10.1017/S0031182011000953 21854702

[B8] Blázquez Martín A (1998) Análisis de *Phlebotomus* (Diptera, Psychodidae) en la alta Extremadura: Taxonomía, distribución y fenología. PhD Thesis, University of Extremadura, Spain.

[B9] Boudabous R Bounamous A Jouet D Depaquit J Augot D Ferté H Berchi S Couloux A Veuille M Babba H (2009) Mitochondrial DNA differentiation between two closely related species, Phlebotomus (Paraphlebotomus) chabaudi and Phlebotomus (Paraphlebotomus) riouxi (Diptera: Psychodidae), based on direct sequencing and Polymerase Chain Reaction-Restriction fragment length polymorphism. Annals of the Entomological Society of America 102(3): 347–353. 10.1603/008.102.0301

[B10] Bounamous A Boudabous R Jouet D Augot D Ferté H Babba H Berchi S Depaquit J (2008) Caractérisation moléculaire et morphologique de deux espèces affines de *Paraphlebotomus*: *Phlebotomuschabaudi* Croset, Abonnenc& Rioux, 1970 et *Ph.riouxi* Depaquit, Killick-Kendrick & Léger, 1998 (Diptera: Psychodidae). Parasite (Paris, France) 15(4): 565–571. 10.1051/parasite/2008154565 19202763

[B11] Bravo-Barriga D Parreira R Maia C Afonso MO Blanco-Ciudad J Serrano FJ Pérez-Martín JE Frontera E (2016) Detection of *Leishmania* DNA and blood meal sources in phlebotomine sand flies (Diptera: Psychodidae) in western of Spain: update on distribution and risk factors associated. Acta Tropica 164: 414–424. 10.1016/j.actatropica.2016.10.003 27720626

[B12] Collantes F Martínez-Ortega E (1997) Sobre la validez taxonómica de *Phlebotomuslongicuspis* (Nitzulescu, 1931) (Diptera: Psychodidae). Boletín de la Asociación Española de Entomología. 21: 141–146.

[B13] Czerny L Strobl G (1909) Spanische Dipteren III. Beitrag. Verhandlungen der kaiserlichköniglichen zoologisch-botanischen Gesellschaft in Wien 59: 121–301.

[B14] Depaquit J Léger N Killick-Kendrick R (1998) Description de Phlebotomus (Paraphlebotomus) riouxi n. sp.(Diptera-Psychodidae) d’Afrique du Nord. Parasite (Paris, France) 5(2): 151–158. 10.1051/parasite/1998052151 9754311

[B15] Di Muccio T Marinucci M Frusteri L Maroli M Pesson B Gramiccia M (2000) Phylogenetic analysis of Phlebotomus species belonging to the subgenus Larroussius (Diptera, Psychodidae) by ITS2 rDNA sequences. Insect Biochemistry and Molecular Biology 30(5): 387–393. 10.1016/S0965-1748(00)00012-6 10745162

[B16] Gállego M Rioux JA Rispail P Guilvard E Gállego J Portús M Delalbre A Bastien P Martínez-Ortega E Fisa R (1990) Primera denuncia de flebotomos (Diptera, Psychodidae, Phlebotominae) en la provincia de Lérida (España, Cataluña). Revista Ibérica de Parasitología 50(1–2): 123–127.

[B17] Gállego Berenguer J Botet J Vynieta MP Gállego M (1992) Los flebotomos de la España peninsular e Islas Baleares: identificación y corología: comentarios sobre los métodos de captura. In memoriam al profesor doctor D. Francisco de Paula Martínez Gómez, 579–600. https://dialnet.unirioja.es/servlet/articulo?codigo=1166628

[B18] García San Miguel L Sierra MJ Vazquez A Fernandez-Martínez B Molina R Sanchez-Seco MP Lucientes J Figuerola J de Ory F Monge S Suarez B Simón F (2021) Phlebovirus-associated diseases transmitted by phlebotominae in Spain: Are we at risk? Enfermedades Infecciosas Microbiología Clínica. Elsevier Doyma. 10.1016/j.eimc.2020.02.026 34353512

[B19] Gil Collado J Márquez FM Marín MS (1989) Los flebotomos en España. Revista de Sanidad e Higiene Pública 63: 15–34. 2699769

[B20] González E Jiménez M Hernández S Martín-Martín I Molina R (2017) Phlebotomine sand fly survey in the focus of leishmaniasis in Madrid, Spain (2012–2014): Seasonal dynamics, *Leishmaniainfantum* infection rates and blood meal preferences. Parasites & Vectors 10(1): e368. 10.1186/s13071-017-2309-z PMC5540423 28764772

[B21] González E Molina R Iriso A Ruiz S Aldea I Tello A Fernández D Jiménez M (2021) Opportunistic feeding behaviour and *Leishmaniainfantum* detection in *Phlebotomusperniciosus* females collected in the human leishmaniasis focus of Madrid, Spain (2012–2018). PLoS Neglected Tropical Diseases 15(3): e0009240. 10.1371/journal.pntd.0009240 PMC7993803 33720936

[B22] Jiménez M González E Iriso A Marco E Alegret A Fúster F Molina R (2013) Detection of *Leishmaniainfantum* and identification of blood meals in *Phlebotomusperniciosus* from a focus of human leishmaniasis in Madrid, Spain. Parasitology Research 112(7): 2453–2459. 10.1007/s00436-013-3406-3 23535889

[B23] Lane RP Alexander B (1988) Sandflies (Diptera: Phlebotominae) of the Canary Islands. Journal of Natural History 22(2): 313–319. 10.1080/00222938800770241

[B24] Lehrter V Bañuls AL Léger N Rioux JA Depaquit J (2017) Phlebotomus (Paraphlebotomus) chabaudi and *Phlebotomusriouxi*: Closely related species or synonyms? Parasite (Paris, France) 24: e47. 10.1051/parasite/2017050 PMC5711378 29194032

[B25] León Sanz CM Collantes F Martínez-Ortega E (1998) Rectificación a la primera cita de Flebotomos (Diptera, Psychodidae) en la Península Ibérica. Graellsia 54(0): e114. 10.3989/graellsia.1998.v54.i0.349

[B26] León Sanz CM Collantes F Martínez-Ortega E (1999) Revisión de la colección Nájera de Flebotomos (Diptera, Psychodidae) depositada en el Museo Nacional de Ciencias Naturales de Madrid. Graellsia 55(0): 217–221. 10.3989/graellsia.1999.v55.i0.330

[B27] Lucientes-Curdi J Benito-de-Martín MI Castillo-Hernandez JA Orcajo-Teresa J (1991) Seasonal dynamics of *Larroussius* species in Aragón (NE Spain). Parassitologia 33: 381–386. https://europepmc.org/article/med/1841232 1841232

[B28] Lucientes-Curdi J Castillo JA Tang Y Benito MI Ferrer-Dufol M Garcia-Salinas MJ Peribañez MA Guarga-Penella JL (1995) Sobre el hallazgo de *Phlebotomusperniciosus* Newstead, 1911 (DipteraPsychodidae) parasitado por *Mastophorusmuris* (Gmelin, 1790) (Nematoda: Spirurina). Zapateri 5: 179–182.

[B29] Marcondes CB (2007) A proposal of generic and subgeneric abbreviations for phlebotomine sandflies (Diptera: Psychodidae: Phlebotominae) of the world. Entomological News 118(4): 351–356. 10.3157/0013-872X(2007)118[351:APOGAS]2.0.CO;2

[B30] Martín-Sánchez J Gramiccia M Pesson B Morillas-Márquez F (2000) Genetic polymorphism in sympatric species of the genus *Phlebotomus*, with special reference to *Phlebotomusperniciosus* and *Phlebotomuslongicuspis* (Diptera, Phlebotomidae). Parasite (Paris, France) 7(4): 247–254. 10.1051/parasite/2000074247 11147032

[B31] Martínez-Ortega E (1986) Biology of Iberian sandflies (Diptera, Psychodidae) under natural conditions. Annali dell’Istituto Superiore di Sanita 22(1): 73–78. https://pubmed.ncbi.nlm.nih.gov/3752827/3752827

[B32] Martínez-Ortega E Conesa Gallego E (1987a) Los flebotomos (Diptera, Psychodidae) del sureste de la Península Ibérica, presentación del hábitat y metodología del muestreo. Mediterránea. Serie de Estudios Biológicos 9: 63–86. 10.14198/mdtrra1987.9.06

[B33] Martínez-Ortega E Conesa Gallego E (1987b) Caracteres morfológicos de interés taxonómico de los flebotomos (Diptera, Psychodidae) de la Península Ibérica. Anales de Biología 11(3): 43–53. https://revistas.um.es/analesbio/article/view/35751

[B34] Martínez-Ortega E Ward RD Martín Luengo F Conesa Gallego E (1982) Nueva distribucion de Phlebotomus (Larroussius) longicuspis Nitzulescu 1930 (Diptera, Phlebotomidae) en España. Revista Ibérica de Parasitología 42: 283–288.

[B35] Martínez-Ortega E Conesa Gallego E Diaz Sánchez F (1988) Aportación al conocimiento de los flebotomos (Diptera, Psychodidade) de las Islas Canarias. Revista Ibérica de Parasitología 48(1): 89–893. https://agris.fao.org/agris-search/search.do?recordID=ES8900049

[B36] Martínez-Ortega E Gallego EC Lozano HR (1996) A new sandfly from Spain: Phlebotomus (Larroussius) langeroni Niztulescu, 1930 (Diptera, Psychodidae). Parasite (Paris, France) 3(1): 77–80. 10.1051/parasite/1996031077

[B37] Medlock JM Hansford KM Van Bortel W Zeller H Alten B (2014) A summary of the evidence for the change in European distribution of phlebotomine sand flies (Diptera: Psychodidae) of public health importance. Journal of Vector Ecology 39(1): 72–77. 10.1111/j.1948-7134.2014.12072.x 24820558

[B38] Morillas-Márquez F Guevara Pozo D (1994) On the validity of the subgenus Phlebotomus (Abonnencius) (Diptera: Psychodidae: Phlebotominae). Research and Reviews in Parasitology 5(1): 55–56.

[B39] Morillas-Márquez F Úbeda Ontiveros JM Guevara Benítez DC González Castro J (1982a) Confirmación de la presencia en España de Phlebotomus (Paraphlebotomus) chabaudi Croset, Abonnenc y Rioux, 1970 (Diptera, Phlebotomidae). Revista Ibérica de Parasitología 42(3): 345–346.

[B40] Morillas-Márquez F Guevara Benítez D. C D. C Gil Collado J Ubeda Ontiveros JM (1982b) Presencia en España de Phlebotomus (Larroussius) longicuspis (Nitzulescu, 1930). Revista Ibérica de Parasitolgía, 191–196.

[B41] Morillas-Márquez F Guevara Benítez DC Úbeda Ontiveros JM González Castro J (1983a) Teratismos observados en *Sergentomyiaminuta* (Rondani, 1843) (Diptera,Phlebotomidae) capturados en España. Revista Ibérica de Parasitología 43(2): 135–143.

[B42] Morillas-Márquez F Castillo Remiro A Ubeda Ontiveros JM (1983b) Existencia de *Sergentomyiafallax* (Parrot, 1921) (Diptera, Phlebotomidae) en las Islas Canarias. In: III National congress of Parasitology, Barcelona.

[B43] Morillas-Márquez F Úbeda Ontiveros JM Castillo Remiro A (1984) Nuevos datos sobre *Phlebotomusfortunatarum* Úbeda Ontiveros y cols, 1982 y presencia de *Sergentomyiafallax* (Parrot, 1921)(Díptera, Phlebotomidae) en el archipiélago Canario. Revista Ibérica de Parasitología 44(1): 29–38.

[B44] Morillas-Márquez F Sanchís Marín MC Martín Sánchez J Acedo Sánchez C (1991) On *Phlebotomusperniciosus* Newstead, 1911 (Diptera, Phlebotomidae) in the province of Almería in southeastern Spain. Parassitologia 33: 437–444. 1841241

[B45] Parrot L (1936) Notes sur Les Phlébotomes XX. – Sur Phlebotomuslangeronivar.longicuspis Nitzulescu, 1930. Archives. Institut Pasteur d’Algerie 14: 137–143.

[B46] Pesson B Ready JS Benabdennbi I Martín‐Sánchez J Esseghir S Cadi‐Soussi M Morillas-Marquez F Ready D (2004) Sandflies of the *Phlebotomusperniciosus* complex: Mitochondrial introgression and a new sibling species of *P.longicuspis* in the Moroccan Rif. Medical and Veterinary Entomology 18(1): 25–37. 10.1111/j.0269-283x.2004.0471.x 15009443

[B47] Remoli ME Jiménez M Fortuna C Benedetti E Marchi A Genovese D Gramiccia M Molina R Ciufolini MG (2016) Phleboviruses detection in *Phlebotomusperniciosus* from a human leishmaniasis focus in South-West Madrid region, Spain. Parasites & Vectors 9(1): 1–11. 10.1186/s13071-016-1488-3 27075742 PMC4831143

[B48] Rioux JA Croset H Leger N (1974) Presence in Spain of *Phlebotomuschabaudi* Croset, Abbonenc and Rioux, 1970 (Diptera - Psychodidae). Annales de Parasitologie Humaine et Comparee 49(4): 505–507. 10.1051/parasite/1974494505 4457035

[B49] Rioux JA Croset H Leger N Maistre M (1975) Comments on the infraspecific taxonomy of *Sergentomyiaminuta* (Rondani, 1843), *S.africana*. Annales de Parasitologie Humaine et Comparee 50(5): 635–641. 10.1051/parasite/1975505635 1078540

[B50] Rioux JA Gallego J Jarry DM Guilvard E Maazoun R Périères J Becqueriaux A Belmonte A (1984) A new *Phlebotomus* for Spain. Phlebotomus (Adlerius) mascittii Grassi, 1908. Annales de Parasitologie Humaine et Comparee 59(4): 421–425. 10.1051/parasite/1984594421 6486628

[B51] Rispail P Léger N (1998) Numerical taxonomy of Old World Phlebotominae (Diptera: Psychodidae): 2. Restatement of classification upon subgeneric morphological characters. Memorias do Instituto Oswaldo Cruz 93(6): 787–793. 10.1590/S0074-02761998000600016 9921303

[B52] Rosado Maestre D (1997) Estudio de flebotomos en Cáceres: taxonomía, distribución y fenología. PhD Thesis, University of Extremadura, Spain. https://dialnet.unirioja.es/servlet/tesis?codigo=202294&info=resumen&idioma=SPA

[B53] Sanbonmatsu-Gámez S (2005) Infección neurológica por virus Toscana en la provincia de Granada: Estudio Clínico-Epidemiológico. PhD Thesis, University of Granada, Spain.

[B54] Sanchez Clemente N Ugarte-Gil CA Solórzano N Maguiña C Pachas P Blazes D Bailey R Mabey D Moore D (2012) *Bartonellabacilliformis*: A systematic review of the literature to guide the research agenda for elimination. PLoS Neglected Tropical Diseases 6(10): e1819. 10.1371/journal.pntd.0001819 PMC3493376 23145188

[B55] Tabbabi A Rhim A Ghrab J Martín O Aoun K Bouratbine A Ready P (2014) Phlebotomus (Paraphlebotomus) riouxi: A synonym of *Phlebotomuschabaudi* without any proven vectorial role in Tunisia and Algeria. Medical and Veterinary Entomology 2(1): 51–59. 10.1111/mve.12067 25171607

[B56] Úbeda Ontiveros JM Morillas-Márquez F (1983) Designation of the holotype of *Phlebotomusfortunatarum* Úbeda Ontiveros et al. 1982 (Diptera, Phlebotomidae). Revista Ibérica de Parasitología 43(3): 307–308. http://bibliotecavirtual.ranf.com/i18n/consulta/registro.cmd?id=11946

[B57] Úbeda Ontiveros JM Morillas-Márquez F Guevara Benítez DC López Román R Cutillas Barrios C (1982) Flebotomos de las Islas Canarias (España). Revista Ibérica de Parasitología, Extra.: 197–206.

